# “At least someone thinks I’m doing well”: a real-world evaluation of the quit-smoking app StopCoach for lower socio-economic status smokers

**DOI:** 10.1186/s13722-021-00255-5

**Published:** 2021-07-28

**Authors:** Eline Meijer, Janneke S. Korst, Kristiene G. Oosting, Eline Heemskerk, Sander Hermsen, Marc C. Willemsen, Bas van den Putte, Niels H. Chavannes, Jamie Brown

**Affiliations:** 1 Public Health and Primary Care, Leiden University Medical Center, Leiden, The Netherlands; 2 National eHealth Living Lab, Leiden University Medical Center, Leiden, The Netherlands; 3 Pharos Dutch Center of Expertise On Health Disparities, Utrecht, The Netherlands; 4 OnePlanet Research Center, Imec NL, Wageningen, The Netherlands; 5 The Netherlands Expertise Centre for Tobacco Control, Trimbos Institute, Utrecht, The Netherlands; 6 Amsterdam School of Communication Research, University of Amsterdam, Amsterdam, The Netherlands; 7 Department of Behavioural Science and Health, University College London, London, UK; 8 SPECTRUM Collaboration, London, UK

**Keywords:** Socio-economic status, Smoking, eHealth, Blended care, Real-world evaluation, Implementation

## Abstract

**Background:**

Smoking is more prevalent and persistent among lower socio-economic status (SES) compared with higher-SES groups, and contributes greatly to SES-based health inequities. Few interventions exist that effectively help lower-SES smokers quit. This study evaluated “De StopCoach”, a mobile phone delivered eHealth intervention targeted at lower-SES smokers based on the evidence-based StopAdvisor, in a real-world setting (five municipalities) in The Netherlands in 2019–2020.

**Method:**

We conducted individual semi-structured interviews with project leaders, healthcare professionals, and participating smokers (*N* = 22), and examined log data from the app (*N* = 235). For practical reasons, SES of app users was not measured. Qualitative data were analysed using the Framework Approach, with the Consolidated Framework for Implementation Research (CFIR) and Unified Theory of Acceptance and Use of Technology (UTAUT) as theoretical models.

**Results:**

Qualitative data showed that factors from the Intervention and Setting domains were most important for the implementation. StopCoach seemed suitable for lower-SES smokers in terms of performance and effort expectancy, especially when integrated with regular smoking cessation counseling (SCC). Key barriers to implementation of the app were limited integration of the app in SCC programs in practice, difficulty experienced by project leaders and healthcare professionals to engage the local community, and barriers to SCC more generally (e.g., perceived resistance to quitting in patients) that prevented healthcare professionals from offering the app to smokers. Quantitative data showed that 48% of app users continued using the app after the preparation phase and pre-quit day, and that 33% of app users had attempted to quit. Both app adherence and quit attempts were more likely if smokers also received SCC from a professional coach. Posthoc analyses suggest that adherence is related to higher likelihood of a quit attempt among participants with and without a professional coach.

**Conclusions:**

Smokers, healthcare professionals and project leaders indicated in the interviews that the StopCoach app would work best when combined with SCC. It also appears from app log that app adherence and quit attempts by app users can be facilitated by combining the app with face-to-face SCC. As such, blended care appears promising for helping individual smokers quit, as it combines the best of regular SCC and eHealth. Further research on blended care for lower-SES smokers is needed.

**Supplementary Information:**

The online version contains supplementary material available at 10.1186/s13722-021-00255-5.

## Background

Despite gradual decline in smoking prevalence in many high-income countries, a substantial proportion of the adult population still smokes [1]. Evidence-based interventions such as pharmacotherapy, and individual or group behavioural smoking cessation counselling (SCC) can increase individual success rates, but most smokers attempt to quit without assistance [2]. The success rate for unassisted quit attempts is only around 5% after 1 year among Dutch smokers, and for individuals with a lower socio-economic status (SES) this percentage is even smaller [3].

Smoking is more prevalent and persistent among lower-SES compared with higher-SES groups, and contributes greatly to SES-based health inequities [4–8]. SES can be defined in several ways [9]. In tobacco research, SES is often defined by educational level, which has been found to be an important indicator of risk of smoking independent of occupational class and income [9]. Compared to their higher-SES counterparts, lower-SES smokers typically are heavier smokers, have more difficulty to quit successfully, and often receive less social support for smoking cessation [10, 11]. In addition, lower-SES smokers are more often hindered in quitting successfully by SES-related problems (e.g., financial debts, housing/family problems), but can be helped to quit by a personalized and proactive approach [12–14]. Most interventions that intend to help smokers quit smoking are more effective among higher-SES than lower-SES smokers [8, 15]. A review showed that, overall, individual-level interventions compared with no support can help lower-SES smokers quit. However, it did not matter for effectiveness whether individual-level interventions were targeted specifically at lower-SES smokers [15], possibly because the community and population level are not sufficiently included. One of the few interventions published that targeted lower-SES smokers is the UK-based eHealth intervention StopAdvisor [16–18]. This website-delivered intervention was based on the PRIME theory of motivation, the incorporation of a range of behaviour change techniques and experience in designing web-based interventions for behaviour change, and extensive user-testing with lower-SES smokers [17, 19]. A randomized controlled trial among 4613 daily smokers showed that, among lower but not higher-SES smokers, StopAdvisor was used more often and resulted in significantly higher abstinence rates than an information-only website [16].

eHealth interventions can help smokers quit more effectively, especially if interventions are personalized, interactive, and include text messages [20, 21]. From a public health perspective, eHealth is promising for reducing smoking prevalence among people who do not (want to) use pharmacotherapy nor receive traditional SCC, given its low thresholds for use. However, eHealth interventions often suffer from low adherence (high drop-out) rates, and are used less often by lower-SES than higher-SES individuals [22, 23]. Blended care, an integration of eHealth and face-to-face treatment, may be the most promising method for helping individual smokers quit [24]. As part of blended care, eHealth can add valuable enhancements to face-to-face behavioural interventions, including availability and accessibility in the smoker’s own environment, tailoring to users’ needs, low costs, and easy scalability [22, 24].

The implementation of eHealth interventions often fails, with shortage of financial resources being an important barrier [25]. The complexity of the implementation process of eHealth interventions is often underestimated [26]. This results in, for example, interventions with low acceptability and feasibility, organizations that are not ready for the intervention, or potential users having personal or professional reasons not to use the intervention. Implementation of blended interventions for smoking cessation likely depends on healthcare professional factors associated with implementation of SCC more generally, such as limited self-efficacy, knowledge or skills, or unbeneficial beliefs—e.g., that smokers are themselves responsible for smoking, or that smokers are not motivated to quit [27–31]. Various theoretical models have been advanced to understand implementation processes. The Consolidated Framework for Implementation Research (CFIR), a widely used synthesis of nineteen frameworks and theories on implementation, proposes five interacting domains that determine implementation success: intervention characteristics (e.g., intervention quality), the outer setting (e.g., external policies) and inner setting in which the intervention is implemented (e.g., implementation climate), characteristics of individuals implementing the intervention (e.g., self-efficacy), and the implementation process (e.g., planning) [32, 33]. Acceptance and use of eHealth interventions can be explained using the Unified Theory of Acceptance and Use of Technology (UTAUT) [34]. UTAUT proposes that performance and effort expectancy (i.e., gains and ease associated with using the technology, respectively) and social influence determine intention, and both intention and facilitating conditions determine actual use. These relations are moderated by gender, age, experience and voluntariness of use.

This study evaluated StopCoach, a mobile phone delivered eHealth intervention (app) targeted at lower-SES smokers based on StopAdvisor, in a real-world setting. We aimed to implement StopCoach in blended care settings within five municipalities in The Netherlands. The Netherlands were chosen as several organizations in the field indicated that they were in need of a smoking cessation intervention for lower-SES smokers [35]. The project team cooperated with five local project leaders who engaged healthcare professionals to implement the intervention among smokers intending to quit. Smokers could use the app regardless of their SES. We conducted individual semi-structured interviews with project leaders, healthcare professionals, and participating smokers and examined quantitative data generated by the app. The following research questions were addressed:

1. What is the experience of project leaders and healthcare professionals implementing StopCoach, which barriers and facilitators emerge from the implementation process, and what is the experience of smokers using StopCoach?
2. Is app adherence to StopCoach related to participant (i.e., municipality), smoking (e.g., number of cigarettes per day) and initial app usage characteristics (e.g., enabling notifications)?
3. What percentage of app users undertake a quit attempt?
4. How do users evaluate their progress in quitting?
5. Are quit attempts related to participant, smoking and initial app usage characteristics?

## Methods

### Design and participants

This real-world study was performed in five municipalities involved in the proof-of-concept implementation project of StopCoach in The Netherlands in 2019–2020. Municipalities were recruited by Pharos. Pharos supports 155 municipalities that participate in the “Healthy in the City” program by the Dutch Ministry of Health, Welfare and Sport in reducing local health disparities. Any interested municipality could take part. Participating municipalities (i.e., Goeree-Overflakkee, Hulst, Roermond, Stadskanaal, and Weststellingwerf) were located in various regions in The Netherlands with different degrees of urbanisation, with numbers of inhabitants ranging from around 26,000–58,000 in 2019. Each municipality appointed their own project leader, who selected a setting in which to implement the app, and involved local healthcare professionals that could offer the app to smokers as part of a SCC program. Project leaders were instructed to take local health and social services into account, such that healthcare professionals could refer smokers for other problems (e.g., housing, financial debts) if necessary. In three municipalities, the implementation project was embedded in ongoing projects that focused on reducing smoking rates among pregnant women in the region, or promoting health among either the municipality inhabitants or the regional population which contained a relatively large share of lower-SES individuals. StopCoach was a stand-alone project in the two other municipalities, where it was implemented in midwifery practices and existing SCC programs serving the broader population of smokers. Smokers could use the app regardless of their SES.

Qualitative data was collected through interviews with five project leaders, seven healthcare professionals and ten participating smokers between November 2019 and February 2020. We aimed to include mostly lower-SES smokers. One project leader also participated as a healthcare professional and was interviewed twice (project leader 1/healthcare professional 4). Healthcare professionals were recruited through project leaders, and smokers were contacted by one of the interviewers after they had indicated that they were open to being interviewed on an information sheet about the project.

Quantitative data was retrieved from all app users (*N* = 235) who indicated that they resided in one of the five municipalities involved, and who used the app during the period when the project was running (i.e., May 2019 through June 2020) regardless of how long they had used the application (see Additional file 1 for descriptive statistics). Ethical approval was provided by Trimbos Institute’s Ethical Committee (2548425).

### Intervention

StopCoach comprised an 8-week stepwise program to support smokers in the initial phase of their quit attempt. The app was based on StopAdvisor [16, 17], and fully complied with Dutch clinical guidelines for smoking cessation and tobacco dependence [36, 37]. We ensured that all behaviour change techniques used in StopAdvisor were incorporated in the Dutch StopCoach app. We involved lower SES smokers and healthcare professionals in several design choices, such as the appearance of the virtual coach, which was an important feature of the app. The virtual coach provided practical and motivational support throughout the process. The application was tested on ease of understanding and accessibility in think-aloud sessions with five lower-SES smokers also reported low literacy, and modified based on their feedback (see Additional file 2 for more details). The app could be downloaded for free.

### Procedure

Qualitative data were collected using in-depth semi-structured individual telephonic interviews (see Additional file 3 for interview protocols). We sent potential participants information about the study’s aims and procedures, processing of personal data and protection of privacy by e-mail. We contacted potential participants 1 week later to discuss any questions regarding the project, and scheduled an interview. Oral informed consent was recorded in a separate audio file. Interviews focused on evaluation of the app in all groups, complemented by experiences during the implementation process and support by Pharos for project leaders; experiences with SCC and the app for healthcare professionals; and smoking history and behaviour, and experiences with blended care for participants. Two interviewers (EH and another Pharos employee) specialized in lower-SES groups conducted most of the interviews. JK, at the time Master student in Medicine, interviewed four smokers. Duration of the interviews was 30 min on average, ranging from 15 to 46 min. Interviews were recorded and transcribed verbatim. The interview recording of healthcare professional 6 was aborted after 12 min due to technical malfunction. All interview participants received a €15 gift voucher.

Quantitative data were obtained from the app. During the installation process of the app, participants were asked to actively agree with the app’s privacy statement. The statement was drafted in understandable Dutch language, as lower literacy is more common among the lower-SES target group. If they agreed, they were asked again in a pop-up window whether they were sure. The privacy statement explained which data were collected, purposes of data collection (e.g., scientific research), and how users could have their data removed (i.e., by sending an e-mail). This procedure suffices the GDPR and Dutch privacy law requirements for consent.

### Measures

Measures had no missing values, unless indicated otherwise.

#### Participant and smoking characteristics

The app asked participants about the municipality participants resided in (self-report with five options, and ‘other municipality’), number of cigarettes smoked per day, smoking within 30 min after waking up (yes/no), previous quit attempts ever (yes/no; no criteria specified for duration), and whether they currently had a professional coach for SCC (yes/no). For cigarettes per day, two values over 80 were recoded into 80 in order to correct for inaccurate data entry [38]. Participants could indicate in the preparation step which reason(s) to quit they had. Participants were not obliged to answer this question (n = 18, 8% missing values). If there was any activity recorded in the preparation step, we also calculated number of reasons to quit reported. Demographic variables including SES were not asked, to prevent putting off app users in the onboarding process.

#### App usage

The app registered whether participants had enabled push-notifications, and had chosen the male/female virtual coach. We calculated duration of app usage (in days) by subtracting the date of last activity from the date of initial activity in the app, and assessed in which step participants showed their last activity within the app, in how many of the steps they had shown activity, and how many activities were registered in the app in total.

### Analysis

#### Qualitative analysis

To answer research question 2, initial qualitative data analysis was performed by JK and KO, and supervised by EM who also performed the final, integrative analytic steps. Analysis took place according to the principles of the framework approach and cross-case analysis [39, 40]. Separate coding trees were constructed for project leaders, healthcare professionals and participants, based on two randomly selected transcripts from each group. We combined inductive and deductive analysis, such that theoretical frameworks guided the analysis, but at the same time we were open to novel themes within the theoretical domains that emerged during the analytic process. The CFIR and UTAUT were used as theoretical frameworks [32–34]. The CFIR facilitated our understanding of factors involved in the implementation process, and the UTAUT was used in addition to CFIR’s intervention domain in order to gain in-depth insight into performance expectancy (i.e., gains attained from using a technology) and effort expectancy (i.e., ease associated with using a technology) with regard to StopCoach. Data were coded and processed by JK and KO using Atlas.TI. Whilst coding, the coding trees were improved when necessary. We ensured reliability of the analysis by having five (three healthcare professionals, two participants) randomly selected transcripts coded independently by JK and KO, after which the coded transcripts were discussed and discrepancies were solved. One coded project leader transcript was reviewed in detail by EM and interpretations were discussed. Relevant quotes were subsequently brought together per code, and compared among interview participants within each group to create a cross-case analysis. We made sure that our analysis was grounded in the data by verifying our interpretations with the overall data from the interviews, and by removing answers to overly suggestive questions. Themes emerging for the within-group analysis were merged in an overall synthesis of findings. Illustrative quotes presented in the Results section were abbreviated for length and clarity.

#### Quantitative analyses

For research question 2, a dichotomous adherence variable was constructed, with the non-adherent category including participants who only did the preparation phase, and adherent participants showing activity after the preparation phase. We performed separate univariable logistic regression analyses, with adherence as the dependent variable and participant, smoking and initial app usage characteristic variables (e.g., enabling push-notifications or not) as independent variables. Dummy variables were created for municipality, with the municipality that most app users resided in (Roermond) as the reference category. Independent variables associated with adherence at *p* < 0.05 were then included in a multivariable logistics regression analysis. We performed a sensitivity analysis without the reasons to quit variables, as these variables had missing values and models were using cases with complete data for variables included in the respective model. Descriptive statistics were estimated to answer research questions 3 and 4. For research question 5, we fit univariable and multivariable models as for research question 2, with quit attempt as the dependent variable.

## Results

### Qualitative results (research question 1)

The CFIR domains ‘Intervention characteristics’, ‘Inner setting and Outer setting’ sections appeared most important for the implementation process and are therefore presented in more detail below. Inner setting and outer setting were combined, as the healthcare professionals’ inner setting was part of the project leaders’ outer setting and vice versa, and one project leader also was involved as healthcare professional. In addition, it was difficult to define the inner/outer setting as a specific place, as the settings in which the StopCoach were implemented varied among municipalities and sometimes changed during the project. Results for the CFIR domains ‘Individual characteristics’ and ‘Implementation process’ are provided in brief towards the end of the Qualitative results section. Participating smokers had much to say about Intervention characteristics and also about blended care, but less about other Setting characteristics, Individual characteristics of those implementing the app (project leaders and healthcare professionals), and the Implementation process, as the factors were less visible to them.

Five healthcare professionals were experienced in providing SCC, and two participated in SCC training in order to take part in the project. One project leader was a current smoker, none of the healthcare professionals currently smoked. Six participating smokers reported a lower educational level and two each a middle (P6, P7) or higher educational level (P1, P9). All but one participant (P8) simultaneously participated in a SCC program. At the time of the interview, five participants had quit smoking successfully (P1, P4, P7, P8, and P9), three had fallen back to their former number of cigarettes after attempting to quit (P6, P10) or cutting down (P3), and two were smoking less than before (P2, P5); see Additional file 4 for more background information.

#### Intervention characteristics

##### Effort expectancy

In this study, effort expectancy focuses on the expected ease (or difficulty) associated with using StopCoach. All ten participants downloaded StopCoach, as well as all but one of both the healthcare professionals and project leaders who were interviewed (one did not like using her phone, one project leader was unable to install the app as she was abroad, but used information about the app provided by Pharos). Interviewees discussed experiences with both installing the app, and using the app. Almost all participants thought the application was easy to install, although some needed help with the installation from other participants in their SCC group or the healthcare professional. In contrast, both healthcare professionals and project leaders indicated that installing the app required too many steps to be taken. All participants started using the app after installation, except for P4 who never used eHealth and believed himself too old for this. Several participants and healthcare professionals also believed that older people may struggle with eHealth, for example P6 said:

“The ones who couldn’t manage the installation weren’t very much at home on their phones either. (…) Well, that a 71-year-old lady doesn’t remember how her phone works... I’d forgive her, right?”

In line with this, healthcare professional 3 mentioned:

“I have indeed had problems with all of my groups. That somebody had to sit next to them and help them step by step. A lot of older people don’t know their phone very well or get a bit confused.”

Healthcare professional 6 only offered the app to participants whom she perceived to have sufficient digital literacy, and linked this to age as well:

“I’ll ask them in advance. See, if it’s really someone older sitting here. I’ll ask them, ‘Do you do a lot on the computer, do you work with apps?’ If they say yes, then it’s fine. If I’ve got somebody that does not have anything to do with such a thing, I don’t go with that.”

With regard to using the app after installation, results showed that most participants, healthcare professionals and project leaders found StopCoach easy to use, and those who commented on the design and lay-out were positive about this (e.g., understandable text, adequate use of visual materials). For example, project leader 4 stated that “it is a clear product, for a clear target group”, and she appreciated “the colours, the lay-out, the use of language and the choice to receive messages, or not”. Many participants did have to learn working with the push notifications containing the messages from the virtual coach, such as P6:

“I had to get used to it for a while. The first time I had chosen Suzanne, indeed. And she said, ‘I sent you a message.’ And I would be like, ‘Okay, where?’ Then I went looking in the app. That was the only thing that was unclear about it.”

This participant seemed to have found out quite quickly by herself, and in general appeared to find the application easy to use. However, she did suggest that perhaps the app could explain where to find the messages. Healthcare professionals believed that the app was suitable for the lower-SES target group, mostly because of the accessible language that was used.

##### Performance expectancy

Performance expectancy concerns the expected gains or positive outcomes (or lack thereof) resulting from using StopCoach. Participants initially opened the application daily, then every other day, and then weekly, in line with the app’s decreasing intensity of contact after the quit date. Participants who continued using the app, as well as healthcare professionals, appreciated the descriptive statistics, support provided by the virtual coach, practical information, and tips. The virtual coach’s messages appeared to increase self-efficacy, such as explained by P8:

“You’re not doing it alone, you’re doing it with your coach. Even if it’s someone virtual. You still have a coach that sends a message every day and says you’re doing well. I think that is very important.”

Similarly, P6 stated that the messages reassured her of being on the right track when she did not receive support from her social environment, and she would think “well, at least someone thinks I’m doing well”. Practical information and tips provided by the app were perceived as useful; for example, P8 stated that:

“The first two days were very difficult. But because of that app, I purchased a lot of tomatoes and those washed carrots. So, every time I felt like smoking, I took a carrot or tomato. And that app had another tip for if you wanted to smoke, you had to say: ‘I don’t want to smoke, I want…’ So I was like, ‘I don’t want to smoke, I want a microwave.’ And that really helped me out a lot.”

As such, this participant found that the practical tips offered by the app helped her to quit smoking successfully. She followed up on the advice to eat something healthy when experiencing an urge to smoke, and she focused on what she wanted instead of smoking (in this case, to buy a microwave instead of cigarettes).

Only one participant called the telephone quit-line associated with the app, but most participants did not feel the need as they simultaneously participated in a face-to-face SCC program. Healthcare professional 5 stated that the quit-line option was good, but that this also was the SCC provider’s responsibility.

The app’s limited duration (8 weeks) emerged as its major downside, as was forwarded by a number of participants, healthcare professionals, as well as project leaders. For example, P9 said:

“After eight weeks, it’s finished at once. All it does afterwards is keep on counting [avoided cigarettes, money saved]. So, either you have to throw it off your phone, or you could receive another message from Suzanne [virtual coach] every two or three weeks, ‘How’s it going?’ It’s just an abrupt ending.”

Healthcare professional 3 recognized this from her SCC groups, and underscored that both the app and face-to-face SCC programs are perceived as too limited in duration:

“Recently, a woman from one of my groups indicated that she was disappointed that the app stopped after eight weeks. She still needed support and I can imagine that. That’s what many people say in my groups after six meetings as well, ‘What a pity that it’s over now.’ They like the app a lot, but the support is getting less and less and then it’s nothing.”

Moreover, participants who relapsed stopped using the app. They were bothered by how the app continued to track cigarettes avoided and money saved as if they were still in the process of quitting smoking, because these statistics were incorrect following their relapse. One of these participants did mention that she appreciated the possibility to start over, and all participants who smoked at the time of the interview indicated that they would use Stopcoach for a future quit attempt.

#### Inner and outer setting

The analysis showed several factors in the inner and outer setting. These were thematically organized in factors related to blended care, problems with engaging the community, and barriers to SCC more generally that also appeared to affect the implementation of StopCoach.

##### Blended care

Most participants and healthcare professionals, as well as some project leaders, agreed that the app would not suffice as a stand-alone solution for most smokers, but should be combined with regular SCC. However, blended care was hardly achieved in practice. project leader 1/healthcare professional 4 believed that blended care would combine the best of regular SCC and the app:

“Even though I really do like it [StopCoach], I think that for people who want to quit smoking, the app alone is not enough. I don’t think that is going to work. You need it in combination with SCC. I think it complements each other very well then.”

Similarly, P9 stated that “With the app alone, you won’t quite make it.”, and healthcare professional 5said that “If you find it really difficult to quit, you could just ignore the app. It’s a nice tool, but you really need to see it as an extra tool”. Healthcare professional 5 perceived the ease of ignoring or forgetting about the app as an additional risk for lower-SES smokers. She perceived lower-SES smokers as ‘sensitive to letting things take their course’, perhaps because of other (SES-related) issues that they need to deal with, whereas with the app ‘the smoker has to take action himself’. In addition, several healthcare professionals worried that other problems that lower-SES smokers may experience would hinder using the app as well as quitting smoking more generally, as explained by healthcare professional 3:

“It could be the biggest pitfall for these people, the stress and the tensions. And in a lot of families, there is so much going on, or there is a divorce, violence, or whatever. And you cannot just take that away.”

Project leader 2 suggested to incorporate a social map of the municipalities’ healthcare services into the app to facilitate referral. Participants as well as healthcare professionals appreciated that the app’s content corresponded with the SCC meetings, which allowed participants to use the app for later reference after a SCC session. Healthcare professional 5 said:

“I noticed that the app tries to provide personal coaching support. The questions we ask people face-to-face or by phone, those are also covered in the app. That’s very good, I think that’s what’s better about the app [compared to other apps].”

However, in contrast to the app’s compatibility with existing SCC programs and the shared belief that blended care would work best, both participants and healthcare professionals stated that the app was only introduced and installed during the first meeting of the SCC programs. It was hardly integrated with subsequent SCC sessions, other than some healthcare professionals informing whether participants were using the application. For example, healthcare professional 1 stated that “we didn’t use it [StopCoach] very actively, actually. Erm, we helped to install it”, and healthcare professional 2 said that “we did almost nothing with it in the counselling [program].” Healthcare professionals underscored that “it is up to the participants if they will start using the app” (project leader 1/healthcare professional 4), “if they are interested in having the app on their phone” (healthcare professional 3), suggesting that they did not perceive stimulating use of the app as part of their role. Healthcare professionals also indicated that they needed to familiarize themselves more with the app before being able to offer blended care, which required time as healthcare professionals walked through the app in the same pace as participants when using the app for the first time. Some Healthcare professionals intended to blend the app with their existing SCC program in the future, now that they had a better understanding of the app. In line with these healthcare professional accounts, one participant (P6) recalled:

“If there were any questions from the group about StopCoach, I would answer them. Well, the counsellor even said, ‘Oh how nice, I’m learning from this too.’ And then she came up with questions about the application too. But that wasn’t my job, so to speak. It might have been nice if she had known the app better than we did before we started.”

One suggestion was made for further development of the app in order to optimize blended care, that is, healthcare professional 1 desired the possibility of monitoring his clients’ progress in the app.

##### Problems engaging the community

Some healthcare professionals mentioned that they received support for implementing the app from the organizations they were working for. For example, healthcare professional 3 explained:

“In our hospital we agreed with the communication department that StopCoach will be promoted through television screens.”

However, other healthcare professionals stated that it was difficult to engage their colleagues, or healthcare professionals from other organizations in implementing the app. Project leaders encountered similar problems in engaging municipal organizations. Although StopCoach was incorporated in broader health-promoting projects in some of the municipalities involved, the extent to which this facilitated implementation varied. Project leader 3 for example noted that “the municipality said that they wanted to become a smoke free municipality. […] But it is not top priority”. Similarly, project leader 1/healthcare professional 4 explained that the monthly meetings for the overarching project did not add much for StopCoach:

“StopCoach is a standard topic at these meetings. […] But most of the time it was only some kind of update about StopCoach, like ‘do we already have the app’ and ‘maybe you could visit the sports clubs as well, instead of only the GP.”

Several Project leaders mentioned that they had experienced difficulty with working around organizations’ hierarchical structures, which led to delays in making decisions. For example, project leader 1/healthcare professional 4 had made arrangements with a non-profit organization for disadvantaged individuals to get involved, but his contact persons were volunteers and could not get their superiors on board, such that he had to find a new setting for implementing the app. Project leader 4 stated that “I had my contact person at the municipality, […] but she had a superior as well”, and “one told me A and the other told me B”. She was able to solve this with the municipality as follows: “We came to the conclusion that it is better to have one spokesperson”. Project leader 3 encountered problems with an organization that initially was involved in the project, but then decided to offer the app to their lower-SES employees on their own. After that, project leader 3 recalled that “they did not return my calls, did not respond to emails”, and like project leader1/healthcare professional 4, project leader 3 decided to find different partners for the project.

In addition to engaging colleagues and organizations, the importance of engaging smokers from the community was underscored by project leader 5 as well, who explained how ex-smokers became involved in promoting the app:

“They [healthcare professionals] started a course, it was a good one. It was a small group in the beginning. But after the course had ended, that group made a lot of promotion, they really became ambassadors. And they have, there is another course right now, but the group has become bigger, so that has been put away in a good way.”

However, in other communities it continued to be difficult to involve smokers in using the app, such as described by project leader1/healthcare professional 4 who had incorporated StopCoach in a series of vlogs about health:

“I am really happy that it has been viewed over 3000 times, but I did not receive any phone call because of that vlog.”

##### Barriers to SCC related to implementation of StopCoach

The analysis also showed a number of barriers to SCC more generally that hampered the implementation of StopCoach. Project leaders noted that some healthcare professionals lost enthusiasm to work with the app during the project, because of perceived resistance in smokers to consider or discuss quitting smoking. Project leader 4 was one of the project leaders who initially encountered enthusiasm for the project among their healthcare professionals, which was driven by their positive attitude toward the app: *“I invited them all. Everybody said yes. Because everybody seemed to be positive about this app.”* However, several project leaders such as project leader 5 saw that healthcare professionals’ initial enthusiasm decreased:

“I noticed that during the pilot the enthusiasm lowered, because it is quite hard to let people…Quitting smoking is something, it is not really acceptable for conversation.”

Similarly, project leader 2 stated that the midwives that he worked with initially offered StopCoach to all of their pregnant patients, but encountered resistance among pregnant smokers whom they had already advised to quit before. For example, healthcare professional 7 who worked in this municipality stated that pregnant smokers “found it very annoying to talk about the smoking again”. The midwives had therefore decided “fair or not, to focus more on the new patients” (project leader 2) in offering the app. Another more general barrier was lack of clarity on referral options for SCC or for psychosocial and socioeconomic problems more broadly, or even a combined, integral approach. Project leader 4 described her confusion as follows:

“I think that the integral approach exists, or is being created by healthcare teams. […] Or somebody that is really working in that neighbourhood. […] But that is on a municipality level. I don’t know if they, for example, stay in contact with the medical part. I do not have a clear picture of that, but that is what I think.”

#### Other CFIR domains

##### Individual characteristics

The analysis showed that implementation was facilitated by healthcare professionals’ and project leaders’ motivation to implement the app, as well as their positive attitudes toward the app. Healthcare professionals’ and project leaders’ positive attitude likely resulted from both positive effort and performance expectancies, and facilitated the implementation, such as explained by project leader 4:

“I think, because I totally support the product [the app], I could sell it with enthusiasm [to the healthcare professionals], to say so, and that I engaged a lot of people [healthcare professionals] this way.”

Furthermore, project leaders’ experience with managing projects and with the healthcare sector emerged as a facilitating factor.

##### Implementation process

Some project leaders mentioned that the delay in the release of StopCoach caused difficulties during the planning phase, and that is was difficult to schedule preparatory meetings with healthcare professionals. Project leaders appreciated the support by Pharos at the start of, and during, the implementation projects, but most did not make use of the possibility to contact project leaders in other municipalities as they perceived these projects as being too different from their own (project leader 5), or because they believed that they did not need it (project leader 4). Project leader 2 stated that “I can imagine that all project leaders could have learned more from each other, from what we have tried and what barriers we encountered”. Finally, project leader 4 mentioned that “I had no idea about what had already been agreed on, and until the day of today, I do not have that information”, as she had replaced another project leader in her municipality halfway during the implementation project. This project leader also seemed to have difficulty getting a clear picture of how SCC and support for other problems common in people with lower-SES, were organized in the municipality.

### Quantitative results

Most participants (*N* = 235) were relatively heavy smokers (80% smoked within 30 min after waking, median number of cigarettes per day 16) and 70% had attempted to quit before. Seventy-seven percent did not have a professional SCC coach but used the app as a stand-alone intervention, and the most cited reason to quit was improving physical condition and energy. With regard to app usage, results showed that 85% of participants had enabled push-notifications and 74% had chosen the female virtual coach. In addition, 48% continued using the app after the preparation phase and pre-quit day, and only 9% completed the final step. In line with this, median duration of app usage was 1 day (see Additional file 1 for participant and app usage characteristics).

#### Explaining app adherence (research question 2)

Univariable logistic regression analyses showed that participants were significantly more likely to be adherent if they received SCC from a professional coach and had enabled push notifications (see Table 1). Only the association between adherence and receiving SCC from a professional coach remained significant in the subsequent multivariable logistic regression analysis.

**Table 1:** Prediction of app adherence and quit attempts: univariable and multivariable logistic regression analyses (*N* = 217–235)

Variable	Category	OR (95% CI)			
		App adherence		Quit attempts	
		Univariable	Multivariable	Univariable	Multivariable
Participant and smoking characteristics					
Municipality	Roermond (*ref.*)	1		1	1
	Goeree-Overflakkee	0.94 (0.52–1.71)		1.20 (0.64–2.22)	1.21 (0.58–2.54)
	Hulst	0.58 (0.21–1.61)		0.60 (0.19–1.90)	0.56 (0.16–2.01)
	Stadskanaal	0.54 (0.26–1.12)		0.34 (0.14–0.85)*	0.55 (0.19–1.55)
	Weststellingwerf	1.27 (0.63–2.54)		1.06 (0.51–2.20)	0.72 (0.30–1.70)
Smokes < 30 min after waking	Yes	1.38 (0.72–2.64)		1.52 (0.74–3.13)	
Previous quit attempt(s)	Yes	1.75 (0.99–3.10)^+^	1.45 (0.77–2.74)	2.09 (1.10–3.96)*	1.62 (0.81–3.24)
Professional coach	Yes	4.33 (2.20–8.52)***	4.06 (1.94–8.50)***	4.75 (2.50–9.03)***	4.55 (2.29–9.06)***
Reason(s) to quit*					
Example for (own) children	Yes	1.41 (0.80–2.50)		1.57 (0.86–2.86)	
Financial	Yes	1.17 (0.65–2.11)		1.31 (0.70–2.42)	
Longevity	Yes	1.67 (0.94–2.97)^+^	0.90 (0.42–1.94)	1.57 (0.86–2.86)	
Physical condition and energy	Yes	1.38 (0.81–2.36)		1.08 (0.61–1.91)	
Other	Yes	1.24 (0.61–2.49)		1.51 (0.73–3.12)	
# Cigarettes per day		1.00 (0.98–1.02)		1.00 (0.97–1.02)	
# Reasons to quit smoking		1.29 (1.03–1.61)*	1.23 (0.93–1.63)	1.22 (0.98–1.53)^+^	
Initial app usage characteristics					
Enabled push notifications	Yes	2.13 (1.01–4.47)*	2.18 (0.92–5.18)^+^	1.66 (0.74–3.73)	
Virtual coach	Female	0.81 (0.45–1.44)		0.79 (0.43–1.45)	

^+^*p* < 0.10, *p < 0.05, **p < 0.01, ***p < 0.001. Adherence was coded [1] for app users with activity on/after their quit day, [0] for app users without activity on/after quit day (only preparation/last day before quitting). Quit attempt was coded [1] for app users who indicated abstinence at some point, [0] for app users who never indicated abstinence. Multivariable model Adherence χ^2^(5) = 25.61, *p* < 0.001, Cox & Snell *R*^2^ = 0.11, Nagelkerke *R*^2^ = 0.15; Quit attempts χ^2^(6) = 30.42, *p* < 0.001, Cox & Snell *R*^2^ = 0.12, Nagelkerke *R*^2^ = 0.17

The multivariable model included 217 participants, as 18 participants had missing values for the reasons to quit variables. In a sensitivity analysis, the model was fit without the two reasons variables (i.e., longevity and number of reasons; *N* = 235). Having a professional coach and having enabled push notifications were significantly associated with app adherence.

#### Quit attempts and evaluation of quit attempt (research questions 3 and 4)

Results showed that 33% of the entire sample reported to have undertaken a quit attempt while using the app (see Additional file 5 for smoking status and evaluation of quit attempt per step). Specifically, 90 participants (38% of the entire sample) answered at least one question about their smoking status, of whom 78 participants (87% of those who answered; 33% of the entire sample) indicated at some point that they were abstinent. Furthermore, of the 78 participants who reported abstinence at some point, 26 (33%) indicated later on that they were smoking again and 52 (67%; or 22% of the entire sample) did not indicate this, suggesting either successful abstinence or unreported relapse.

Most participants who answered the progress questions (n = 86, 37%) evaluated their quit attempt positively (i.e., good or OK), with the proportion of participants for whom quitting was more difficult (i.e., bad or mediocre) decreasing over time. It appears that participants were more inclined to adhere to the app and answer these questions if quitting smoking went well, and reversely that smokers who found it more difficult to quit dropped out.

#### Explaining quit attempts (research question 5)

Univariable logistic regression analyses showed that participants were significantly more likely to attempt to quit if they had attempted to quit in the past or had a professional coach, and less likely if they lived in Stadskanaal (vs. Roermond), see Table 1. Only having a professional coach remained significant in the multivariable model.

#### Posthoc analysis

We explored associations between adherence and quit attempts among those with and without a professional coach. In both groups, Mann–Whitney tests showed that those who attempted to quit had used the app longer, had activity in more steps of the app, and had more registered activities in the app (all *p*s < 0.001). Results were similar when these associations were examined only among those who were adherent, i.e. who showed activity on, and possibly after, their quit day.

## Discussion

This real-world study evaluated StopCoach, a smartphone application intended to support lower-SES smokers in quitting smoking, in an implementation project in five Dutch municipalities with 235 participating smokers. In addition, we investigated app adherence and quit attempts among app users. Qualitative results, based on individual interviews with local project leaders, healthcare providers and participating smokers suggest that the implementation process is primarily subject to factors related to the intervention itself and the setting in which the intervention is implemented. We found that the implementation was facilitated by project leaders’ and healthcare professionals’ positive attitudes towards the app, which seemed to result largely from positive effort and performance expectancies. That is, they perceived the app as easy to use and useful specifically for the lower-SES target group. This was reflected in participants’ accounts as well, for example, participants appreciated the practical tips and social support provided by the virtual coach, which felt genuine despite their awareness that the coach was virtual. Notably, although the study was not designed to detect SES differences in evaluation of the app, we found that these evaluations were similar among lower, middle, and higher SES smokers. Given that lower-SES smokers typically are less supported in quitting by their social environment than higher-SES smokers [10, 11], eHealth interventions that use messages from a virtual coach to support participants are promising. Supporting this, quantitative results showed that people who had disabled push-notifications, which contained the messages from the coach, were more likely to drop out.

Overall, 33% of app users reported abstinence at some point, indicating that they had undertaken a quit attempt while using the app. Exploratory analyses indicated that participants who were more adherent to the app (i.e., longer use, and more activity in the app) were more likely to attempt to quit, suggesting that the app facilitated their smoking cessation process. It also seemed that participants who lapsed or relapsed were more likely to stop using the app, given that most participants who kept using the app reported that they had not smoked and that quitting went quite well. The current study design does not allow for assessing whether adherence led to quit attempts or vice versa. The app was perceived as less useful for dealing with lapse or relapse, as indicated by some smokers who had stopped using the app after having resumed smoking. In contrast to StopAdvisor, StopCoach allowed smokers to continue using the app after they had smoked, but the motivational messages, information about lapses, and the possibility to restart the app after a lapse do not seem sufficient to ensure that smokers who had smoked kept using the app. StopCoach was also felt to be less useful for facilitating long-term abstinence, as several participants and healthcare professionals found the app’s 8-weeks duration too short. A main barrier to implementation that emerged from the analysis of ‘Intervention’ factors were difficulties installing the app encountered by smokers with limited digital literacy. This occurred despite elaborate testing with low literate individuals and the general finding that the app was experienced as accessible and understandable, and suggests that developing inclusive eHealth interventions remains is challenging [24].

With regard to ‘Setting’ factors, we found that the implementation was facilitated by good compatibility between the app and existing SCC programs, which allowed for blended care [24]. In addition, project leaders, healthcare professionals and participants reported positive attitudes toward blended care for smoking cessation. Blended care was perceived as promising for lower-SES smokers in particular, as adhering to an app requires a pro-active attitude from the smoker, which could be complicated by other problems that lower-SES smokers may experience [12, 13]. Quantitative results also showed that adherence to StopCoach was better among smokers who used the app alongside regular SCC, and that those who were supported by a professional SCC coach were more likely to attempt to quit. At the same time, the implementation of the app in a blended setting appeared hindered by healthcare professionals’ insufficient preparation and familiarity with the app, and possibly perceptions that integrating the app with their existing SCC program was not part of their role [26]. However, since even the limited integration of the app with regular SCC programs observed in the current study was associated with better app adherence and quit attempts, blended care seems a promising route to dealing with the problem of attrition that is common in eHealth interventions for smoking cessation and eHealth more generally [22], as well as to improving smoking cessation outcomes compared to stand-alone eHealth. In order for blended care to succeed, current results suggest that healthcare professionals need to be sufficiently prepared and feel responsible for integrating eHealth into their treatments [26]. Furthermore, blended care is likely to be facilitated if the intervention has specific functionalities for the healthcare professional, such as a mode in which they can quickly walk through the app when preparing blended care, and a monitoring function that allows them to keep track of their patients’ progress. Notably, although blended care seems the most promising route to support people in smoking cessation, a substantial group of smokers in the larger population prefers to quit smoking without formal assistance [41]. Arguably, using a stand-alone eHealth intervention is more beneficial than quitting without any support, and stand-alone eHealth interventions can make important contributions to public health. A stepped care model can be used, such that smokers who fail to quit smoking with a stand-alone eHealth intervention are stimulated to seek professional help. Importantly, matched care—in which smokers are directly referred to treatment that optimally meets their needs—is most appropriate for smokers for whom quitting is urgent (e.g., pregnant smokers, or smokers with smoking-related disease) as well as for smokers experiencing complicating factors (e.g., psychiatric disorders, socioeconomic problems) that they urgently need help with [36].

An important barrier emerging from the analysis of ‘Setting’ factors was the difficulty experienced by project leaders and healthcare professionals to engage colleagues, organizations, the municipality and smokers in the project. This resulted, at least partially, from suboptimal communication at various levels. As a consequence, much of the work had to be done by a small number of people, which threatens sustainability of the implementation [26, 42]. Barriers to providing SCC more generally also played a role, such that the implementation of StopCoach was hindered by HCP’s perceived resistance of patients to discuss smoking and smoking cessation, and insufficient referral networks both for SCC and other problems that lower-SES smokers may have [28, 29]. A randomized controlled trial showed that a functional referral system for services in social domains (e.g., for employment, food, literacy) can facilitate successful quitting, provided that smokers use the referral, underscoring the importance of improving these structures if they are not in place [12].

This study has limitations. First, selection bias likely plays a role in the interview sample for healthcare professionals and participants. Most project leaders stated that it was difficult to enthuse healthcare professionals for working with StopCoach for a longer period of time, but the healthcare professionals included in this study were positive about the project. Relatedly, one municipality was not represented by healthcare professionals, as the implementation process lagged behind and healthcare professionals were not yet involved when the interviews were conducted. Likewise, participating smokers were recruited from three municipalities only. App data furthermore showed that most people using the app did not have a professional helping them quit, whereas all but one of the interview participants participated in SCC and were advised to use the app by their healthcare professional. However, all project leaders were interviewed and provided their views on the implementation process, and we used both qualitative and quantitative data, which reduces risks associated with selection bias and helps provide a representative answer to the research questions. Second, although we assessed a number of smoking characteristics during the installation process of the app, we had to be selective in which variables to measure to prevent putting off app users. As such, the exact proportion of lower-SES smokers in the quantitative sample is unknown as SES was not asked (the same holds for age, gender etcetera, but key smoking characteristics were assessed). This also means that we were unable to assess associations between SES and both adherence and quit attempts. With regard to the interviews, a small majority of participating smokers (6/10) had a lower-SES, but the sample also contained a number of middle and higher SES participants, reflecting the real-world nature of this study. Although the study was not designed to study SES-based differences in evaluation of the app, we did not observe these either among our ten participant interviewees. Pending further research, this suggests that StopCoach may be useful for smokers regardless of SES, despite the fact that it was developed for the lower-SES target group. This corresponds with research showing that higher-SES individuals, like people with lower-SES, prefer simple language [43]. Finally, low app adherence prevented thorough analysis of the smoking cessation process. As noted, low adherence is a well-known problem for eHealth interventions [22]. The current study adds to the literature by offering explanations for low adherence and providing directions for reducing attrition from eHealth interventions for smoking cessation in the future. The real-world setting used in the current study allows for high ecological validity of these results [22].

## Conclusions

This real-world study demonstrated that it is possible to develop an accessible and supportive smoking cessation app for lower-SES smokers. Results based on interviews with project leaders, healthcare professionals and smokers suggest that future eHealth interventions can be made useful for lower-SES smokers by providing practical guidance using understandable language and visual material, including feedback on progress made, and incorporating a virtual coach that sends motivational messages which address the user by name. In addition, such interventions ideally last longer than 8 weeks, and provide adequate and tailored support for smokers who lapsed or relapsed. The implementation of an eHealth smoking cessation intervention in local settings proves to be a complex process, in which intervention characteristics and setting characteristics seem to play a key role. Given that the implementation of many eHealth interventions fails, the current study provides important insight into factors that can facilitate and obstruct successful implementation. Blended care appears to be both challenging and promising, as it requires effort on behalf of the healthcare professionals, as well as smokers willing to participate in both regular SCC and an eHealth intervention, but at the same time can increase adherence to the app and facilitate quit attempts. We believe that, for individual smokers, blended care combines the best of both face-to-face and digital coaching in helping smokers quit smoking successfully.

## Supplementary Information

**Additional file 1.** Descriptive statistics app users and app usage.

**Additional file 2.** Intervention “De StopCoach”.

**Additional file 3.** Interview protocols.

**Additional file 4.** Background information interview participants.

**Additional file 5.** Smoking status and evaluation of progress.

## Data Availability

The datasets used and/or analyzed during the current study are available from the corresponding author on reasonable request.

## Abbreviations

CFIR: Consolidated Framework for Implementation Research
P: Participant (participating smoker)
SCC: Smoking cessation care
SES: Socio-economic status
UTAUT: Unified Theory of Acceptance and Use of Technology
