# Supplementary material for: “At least someone thinks I’m doing well”: a real-world evaluation of the quit-smoking app StopCoach for lower socio-economic status smokers
Source: Addict Sci Clin Pract. 2021 Jul 28;16:48. doi: 10.1186/s13722-021-00255-5 (PMC8320182; doi:10.1186/s13722-021-00255-5)
Supplement: Supplementary file 1 — Additional file 1. Descriptive statistics app users and app usage. [file 13722_2021_255_MOESM1_ESM.docx]

**Additional file 1. Descriptive statistics app users and app usage**

Table S1. Descriptive statistics app users (*N* = 235).

| **Variable** | **Category** | **n (%)** |
| --- | --- | --- |
| Municipality | Goeree-Overflakkee | 58 (24.68) |
|  | Hulst | 17 (7.23) |
|  | Roermond | 85 (36.17) |
|  | Stadskanaal | 37 (15.74) |
|  | Weststellingwerf | 38 (16.17) |
| Smokes <30 minutes after waking | Yes | 189 (80.43) |
| Previous quit attempt(s) | Yes | 164 (69.79) |
| Professional coach | Yes | 54 (22.98) |
| Reason(s) to quit* |  |  |
| Example for (own) children | Yes | 70 (29.79) |
| Financial | Yes | 64 (27.23) |
| Longevity | Yes | 70 (29.79) |
| Physical condition and energy | Yes | 98 (41.70) |
| Other | Yes | 38 (16.17) |
| **Variable** | ***M (SD)*** | **Median (Range)** |
| # Cigarettes per day | 17.73 (11.32) | 16 (0-80) |
| # Reasons to quit smoking | 1.57 (1.27) | 1 (0-5) |
| * Reason(s) to quit had 18 missing values, percentages are valid percentages for 217 participants. | | |

Table S2. Descriptive statistics app usage (*N* = 235).

| **Variable** | **Category** | **n (%)** |
| --- | --- | --- |
| Enabled push-notifications | Yes | 198 (84.26) |
| Virtual coach | Male | 62 (26.38) |
|  | Female | 173 (73.62) |
| Last step with activity | Preparation | 103 (43.83) |
|  | Pre-quit day | 20 (8.51) |
|  | Quit day | 13 (5.53) |
|  | Step 1 (day 1) | 9 (3.83) |
|  | Step 2 (day 2) | 6 (2.55) |
|  | Step 3 (day 3) | 5 (2.13) |
|  | Step 4 (day 4) | 7 (2.98) |
|  | Step 5 (day 5) | 3 (1.28) |
|  | Step 6 (day 6) | 0 (0.00) |
|  | Step 7 (day 7) | 13 (5.53) |
|  | Step 8 (day 10) | 5 (2.13) |
|  | Step 9 (day 12) | 2 (0.85) |
|  | Step 10 (week 3) | 9 (3.83) |
|  | Step 11 (week 4) | 5 (2.13) |
|  | Step 12 (week 5) | 9 (3.83) |
|  | Step 13 (week 6) | 3 (1.28) |
|  | Step 14 (week 7) | 3 (1.28) |
|  | Step 15 (week 8) | 20 (8.51) |
| **Variable** | ***M (SD)*** | **Median (Range)** |
| Duration app usage (days) | 16.97 (36.46) | 1 (0-242) |
| # Steps with activity | 3.85 (4.37) | 2 (1-18) |
| # Registered activities in app | 22.90 (28.58) | 11 (1-171) |
