# Supplementary material for: “At least someone thinks I’m doing well”: a real-world evaluation of the quit-smoking app StopCoach for lower socio-economic status smokers
Source: Addict Sci Clin Pract. 2021 Jul 28;16:48. doi: 10.1186/s13722-021-00255-5 (PMC8320182; doi:10.1186/s13722-021-00255-5)
Supplement: Supplementary file 2 — Additional file 2. Intervention “De StopCoach”. [file 13722_2021_255_MOESM2_ESM.docx]

**Additional file 2. Intervention “De StopCoach”**

StopCoach could be downloaded for free from the App Store (iOS) and Google Play Store. Before starting with the program itself, users were asked to indicate in which municipality they lived (i.e., all five participating municipalities were listed, as well as ‘other municipality’ after which a disclaimer was presented stating that the app was still being evaluated), choose a female (Suzanne) or male (Bas) virtual coach, provide a limited number of smoking and sociodemographic characteristics (see Table 1), and select a quit date at least two days in the future to allow for preparation of the quit attempt. Subsequent steps included a preparation phase, sessions for the last day before quitting and the quit day, daily sessions during week 1 post quit date, two sessions in week 2 post quit date, and six weekly sessions in weeks 3 through 8. Each step provided relevant tips and information in simple, concise language. Visual and audio materials such as videos, voice clips and pictures were used as much as possible to reduce the amount of text in the app.

From the quit date onwards, users were asked whether they had smoked (yes/no) and how they were doing, with answer options (good/OK/mediocre/bad) represented by four smileys. The virtual coach also sent motivational messages (adjusted to users’ answers to the two questions), tips, information, personal stories, and videos for eight weeks through push notifications. Users were personally addressed by their (user) name in these messages. The frequency of the push notifications decreased over the weeks, from daily, to every other day, to once a week. In addition, the app contained a “tips & exercises” section that provided more elaborate thematic information (e.g. on lapse, nicotine replacement therapy, dealing with stress resulting from SES-related problems) and several exercises (e.g. dealing with irritability or with smokers in the social environment). Participants were encouraged to contact their general practitioner or a local social service team if they required help for socioeconomic problems. The application provided easy access to a national telephone quit-line to ask questions or to ask for advice. Users could earn up to five stars by going through the different steps in the application, opening messages and staying smoke-free. In addition, they could easily share their accomplishments with other people through social media platforms such as WhatsApp. During and after the eight weeks of the program, the application provided descriptive statistics on the number of cigarettes not smoked, number of smoke-free days and amount of money saved.


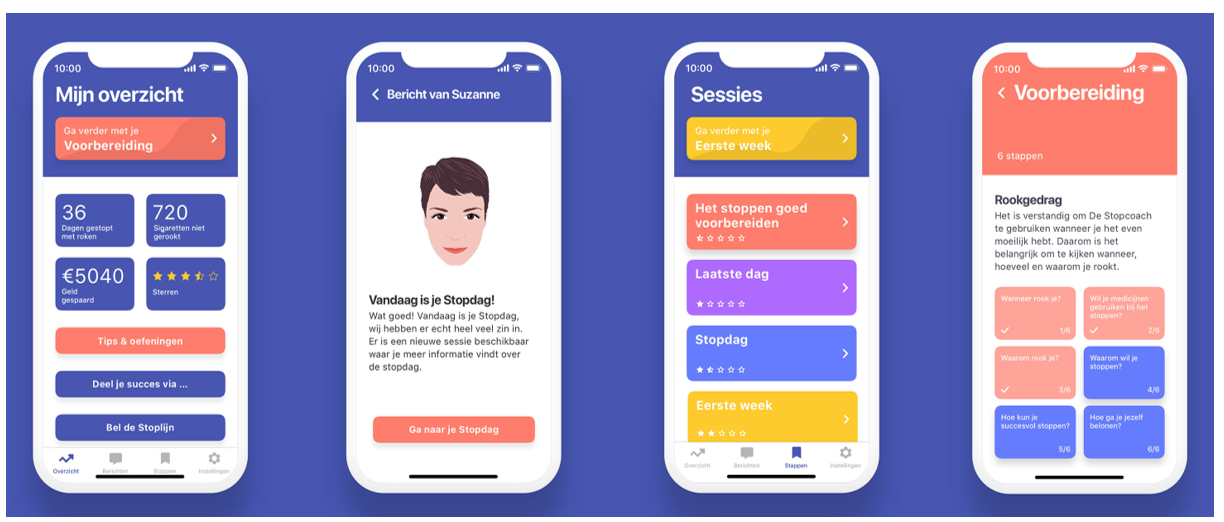
*Screenshots of StopCoach. From left to right: overview page (including statistics for cigarettes avoided and money saved, stars earned, and links to tips & exercises, social media (to share success), and telephone quit-line); message from coach (Suzanne); sessions main menu; sub steps within a session.*
