# Supplementary material for: “At least someone thinks I’m doing well”: a real-world evaluation of the quit-smoking app StopCoach for lower socio-economic status smokers
Source: Addict Sci Clin Pract. 2021 Jul 28;16:48. doi: 10.1186/s13722-021-00255-5 (PMC8320182; doi:10.1186/s13722-021-00255-5)
Supplement: Supplementary file 3 — Additional file 3. Interview protocols. [file 13722_2021_255_MOESM3_ESM.docx]

**Additional file 3. Interview protocols**

Project leaders

1. Background
   1. In which municipality are you involved in this pilot, as a project leader?
   2. What is your profession? How long have you been doing this?
   3. What was the reason your municipality wanted to participate in this pilot?
2. Evaluation implementation within municipality
   1. How did you experience being a project leader? How does that fit in to your profession?
   2. What are your experiences in the preparation of this pilot?
   3. How do think about the start of this pilot?
   4. How did you select the members for the core team?
   5. Did the collaboration work out within the core team?
   6. In what ways did you engage the local healthcare professionals to this pilot? What kind of health care professionals are they? How did you choose them?
   7. How did you promote the app among smokers in your municipality?
   8. What are the experiences in your municipality with recruiting low educated smokers?
   9. What kind of support do the lower educated people in your municipality need to quit smoking and to stay free of smoking? How is this organised in your municipality?
   10. One of the main pillars of this study is to help people with a lower educational level, need more help with underlying factors, to be able to quit smoking. You could think of financial debts, housing problems or difficulties raising their children. What are your thought about this topic? What do people with a lower SES/educational level need, according to you?
   11. How did your municipality organize providing this help for these people?
   12. Do you have any recommendations to improve implementation in your municipality? Do you know any way to reach more people/smokers in your municipality?
3. StopCoach app
   1. Have you seen/downloaded the app yourself? (If no: why not?)
   2. What do you think is positive/good about the app? Could you give some examples?
   3. What do you this is not good/negative about the app? Could you give some examples?
   4. Do you have any suggestions, so that we could improve the app?
4. Evaluation support by Pharos
   We would like to know how you experienced the support by Pharos. We appreciate honest answers. If you weren’t happy with the collaboration, we would like to hear why. Only with honest answers we can try to improve our support as much as possible.
   1. What are your thoughts about the collaboration with Pharos?
   2. How often did you contact each other? How did you experience this contact?
   3. Did you receive information from Pharos about the pilot and the app? What did you think about the information?
   4. How did you like the support given by Pharos? And the advices you got from them?
   5. What could Pharos do to improve the support to the project leaders?
5. Evaluation learning network
   1. How did you experience the collaboration between the municipality’s?
6. Did you smoke in the past, or are you currently smoking?
7. Thank you for participating in this interview. Is there anything else you would like to mention?

Healthcare professionals

1. Background
   1. In which municipality do you work?
   2. What is your function?
   3. What role do you play in smoking cessation counselling in general?
   4. Have you done any training course for smoking cessation counselling?

If yes: what did you do, when was it?

Are you qualified to provide smoking cessation counselling?

1. Evaluation of own role in the pilot

*Standard follow-up question: What went well and what could be improved?*

- 1. How did you get involved in this pilot?
  2. What was your motivation to participate in the pilot?
  3. What role do you play in the guidance of smokers who want to participate in the pilot?
  4. How did you reach/motivate participants from the target group to participate in the pilot?
  5. How did the smokers react to this?
  6. Approximately how many participants have you guided in this pilot to quit smoking in combination with the application StopCoach?
  7. Which part of these participants do you think has a lower education level? (e.g. as a percentage?)

1. Evaluation of implementation in the municipality

*Standard follow-up question: What went well and what could be improved?*

- 1. How was the collaboration with the project leader in your municipality? How was the support from the project team?
  2. What are your experiences in the preparation of this pilot?
  3. How did the start of the pilot go?
  4. How was the collaboration with other (healthcare) professionals in your municipality? Did you share knowledge or information and if so, how?
  5. In your opinion, what guidance do lower educated smokers need to quit smoking and keep it up? How is this organised in your municipality? What could be improved?
  6. An important starting point for the pilot is that lower educated smokers need guidance in underlying problems in order to be able to quit smoking. This concerns, for example, debts, housing problems, or parenting problems. What do you think lower educated smokers need regarding this?
  7. How is the support for underlying problems organised in the pilot in your municipality?
  8. What are your recommendations for further implementation within the municipality? How can we reach more people in your municipality to quit smoking?

1. StopCoach application
   1. Have you seen/installed the application StopCoach yourself?

(If no: why not? Proceed to 6)

- 1. What do you like about the StopCoach app? Can you give examples?
  2. What do you dislike about the StopCoach app? Can you give examples?
  3. Is the app suitable for the target group with a lower education level? Why yes/no?
  4. How could we improve the application?
  5. What are you experiences with offering the application in combination with face-to-face guidance?

1. What have you learned from this pilot?
2. Have you smoked yourself, or do you smoke?
3. Are there any other things you would like to say or ask about the pilot?

Smokers

1. Background
   1. Which municipality do you live in?
   2. What is your year of birth?
   3. What education (level) did you finish?
   4. Are you working at the moment? What is your profession?
2. Smoking cessation
   1. What was your reason/motivation for trying to quit smoking?
   2. Did you try to quit smoking?

If yes: How did this go? For how long have you not smoked? Are you smoking at the moment? What helps you to quit smoking?

If no: What made it difficult for you to quit smoking?

- 1. Have you ever tried to quit smoking before?

If yes: How many times have you tried? How did it go?

1. Smoking cessation counselling
   1. Did you receive help to quit smoking? If yes:
      1. How was the counselling found? Was the counselling in a group or individual? How many meetings were there? Is there a follow-up appointment?
      2. What did you like about the counselling? Can you give examples?
      3. What did you dislike about the counselling? Can you give examples?
2. StopCoach application
   1. Who asked you to use the StopCoach app? What did you think of this? Was the information clear?
   2. Did you download the StopCoach app? How did the installation go?
   3. Approximately how long and how often have you used the StopCoach app?
   4. What do you like about the the StopCoach app? Can you give examples?
   5. What do you dislike about the the StopCoach app? Can you give examples?
   6. How could we improve the app?
   7. Would you recommend the app to other people? Can you explain why?
   8. Have you used other apps or websites while using the the StopCoach app? If yes:
      1. Which ones?
      2. What are your experiences with these?
   9. Have you used medication to help to stop smoking while using the app De StopCoach? If yes:
      1. What did you use? Do you know what this is called?
      2. What are your experiences with this?
3. How can we reach more people in your municipality to quit smoking?
4. Are there any other things you would like to say or ask about StopCoach or smoking cessation in general?
