# Supplementary material for: “At least someone thinks I’m doing well”: a real-world evaluation of the quit-smoking app StopCoach for lower socio-economic status smokers
Source: Addict Sci Clin Pract. 2021 Jul 28;16:48. doi: 10.1186/s13722-021-00255-5 (PMC8320182; doi:10.1186/s13722-021-00255-5)
Supplement: Supplementary file 4 — Additional file 4. Background information interview participants. [file 13722_2021_255_MOESM4_ESM.docx]

**Additional file 4. Background information interview participants**

**Project leaders**

PLs were three females and two males, mean age 42 years (range 29-57). Their main professions were lifestyle coach, project leader for one of the regional health-promoting programs mentioned above, or project manager in addiction care or healthcare more generally. Two PLs were never smokers, two were ex-smokers, and one was a current smoker. All PLs were interviewed such that all municipalities were represented in the sample. PL1 also participated in the project as a HCP and was interviewed in that role as well (PL1/HCP4).

**Healthcare professionals**

HCPs were four females and three males, mean age 50 years (range 28-66). HCPs included a personal fitness trainer, social worker, pulmonary nurse, life coach, SCC coach, nurse practitioner, and a midwife. Five of them had years of experience in providing SCC, one had been providing SCC for a few years, and two participated in SCC training in order to take part in the project. Four HCPs were registered in the national quality register for SSC providers. Six HCPs had never smoked, one was an ex-smoker who quit eight years ago. Furthermore, one and three municipalities were represented by one and two HCPs, respectively, but no HCPs from the fifth municipality were interviewed as HCPs in this community were not yet sufficiently involved in the project when the interviews were conducted (specific municipality removed to prevent traceability of interview participants).

**Participants**

Participants were eight females and two males, aged 52 years on average (range 24-69), of whom six reported a lower educational level and two each a middle or higher educational level. Although not specifically asked, seven participants reported medical diagnoses (e.g. COPD, asthma) and three reported being under high levels of stress. All participants had undertaken one or more quit attempts in the past, and all but one participant (P8) simultaneously participated in a SCC program. At the time of the interview, five participants had quit smoking successfully (P1, P4, P7, P8, and P9), three were back at their former number of cigarettes after quitting (P6, P10) or cutting down (P3), and two were smoking less than before, either by cutting down (P2) or after a quit attempt (P5). Participants from three municipalities were interviewed.
