# Supplementary material for: “At least someone thinks I’m doing well”: a real-world evaluation of the quit-smoking app StopCoach for lower socio-economic status smokers
Source: Addict Sci Clin Pract. 2021 Jul 28;16:48. doi: 10.1186/s13722-021-00255-5 (PMC8320182; doi:10.1186/s13722-021-00255-5)
Supplement: Supplementary file 5 — Additional file 5. Smoking status and evaluation of progress. [file 13722_2021_255_MOESM5_ESM.docx]

**Additional file 5. Smoking status and evaluation of progress**

Table S3. Smoking status and evaluation of progress per app phase: percentages (*N* = 235).

| **App phase** | **Smoking status** | |  | | **Evaluation quit attempt progress (per protocol)** | | | | | |  |
| --- | --- | --- | --- | --- | --- | --- | --- | --- | --- | --- | --- |
|  | ***n (% of participants still using the app)*** | ***% Abstinent (per protocol)*** | | ***% Abstinent (intent to treat)*** | | ***n (% of participants still using the app)*** | ***% Bad*** | ***% Mediocre*** | ***% OK*** | ***% Good*** | |
|  |  |  | |  | |  |  |  |  |  | |
| Step 1 (day 1) | 56 (57) | 88 | | 21 | | 52 (53) | 9 | 31 | **43** | 17 | |
| Step 2 (day 2) | 53 (59) | 89 | | 20 | | 50 (56) | 6 | 32 | **36** | 26 | |
| Step 3 (day 3) | 41 (49) | 85 | | 15 | | 40 (48) | 10 | 20 | **50** | 20 | |
| Step 4 (day 4) | 40 (51) | 93 | | 16 | | 40 (51) | 10 | 15 | **50** | 25 | |
| Step 5 (day 5) | 34 (47) | 88 | | 13 | | 34 (47) | 9 | 12 | **56** | 24 | |
| Step 6 (day 6) | 37 (54) | 78 | | 12 | | 33 (48) | 9 | 15 | **45** | 30 | |
| Step 7 (day 7) | 37 (54) | 78 | | 12 | | 33 (48) | 9 | 15 | **45** | 30 | |
| Step 8 (day 10) | 28 (50) | 89 | | 11 | | 28 (50) | 4 | 18 | 25 | **54** | |
| Step 9 (day 12) | 25 (49) | 84 | | 9 | | 25 (49) | 12 | 8 | **40** | **40** | |
| Step 10 (week 3) | 33 (67) | 79 | | 11 | | 33 (67) | 12 | 12 | **39** | 36 | |
| Step 11 (week 4) | 23 (58) | 96 | | 9 | | 22 (55) | 0 | 18 | **55** | 27 | |
| Step 12 (week 5) | 24 (69) | 75 | | 8 | | 24 (69) | 8 | 25 | **33** | **33** | |
| Step 13 (week 6) | 16 (62) | 81 | | 6 | | 15 (58) | 13 | 20 | **33** | **33** | |
| Step 14 (week 7) | 12 (52) | 83 | | 4 | | 12 (52) | 17 | 8 | 33 | **42** | |
| Step 15 (week 8) | 18 (90) | 78 | | 6 | | 18 (90) | 11 | 11 | 28 | **50** | |
| *Note.* For abstinence, per protocol refers to participants who answered the smoking status, intent to treat refers to all 235 participants (assuming that those who did not answer were smoking at that point). For evaluation of quit attempt progress, the percentage for the most frequently provided answer is printed in bold. Smileys were used to visually represent the answer categories. | | | | | | | | | | | |
